# Factors associated with access to assistive technology and telecare in home-dwelling people with dementia: baseline data from the LIVE@Home.Path trial

**DOI:** 10.1186/s12911-021-01627-2

**Published:** 2021-09-15

**Authors:** Nathalie Genevieve Puaschitz, Frode Fadnes Jacobsen, Janne Mannseth, Renira Corinne Angeles, Line Iden Berge, Marie Hidle Gedde, Bettina Sandgathe Husebo

**Affiliations:** 1 Centre of Care Research (West), Western Norway University of Applied Sciences (HVL), 5009 Bergen, Norway; 2 Centre for Elderly and Nursing Home Medicine (SEFAS), Department of Global Public Health and Primary Care, University of Bergen, Bergen, Norway; 3 VID Specialized University, Stavanger, Norway; 4 NORCE Norwegian Research Centre, Department of Social Science and Health Research, Health Services and Health Economics Research Group, Bergen, Norway; 5 NKS Olaviken Gerontopsychiatric Hospital, Askøy, Norway; 6 Haraldsplass Deaconness Hospital, Bergen, Norway; 7 Municipality of Bergen, Bergen, Norway

**Keywords:** Assistive technology, Telecare, Dementia, Home-dwelling, Caregivers

## Abstract

**Background:**

There is a knowledge gap regarding factors that may influence the access to different devices for home-dwelling people with dementia (PwD). The aim of this study was to identify different assistive technology and telecare (ATT) devices installed in the home and key factors associated with access to such technology.

**Methods:**

The baseline data came from the LIVE@Home.Path trial, a 24-month multi-component intervention including PwDs and their informal caregivers (dyads) and were collected through semi-quantitative questionnaires in three Norwegian municipalities between May and November of 2019. Regression models were applied to detect demographic and clinical factors associated with access to ATT.

**Results:**

Of 438 screened dyads, 276 were included at baseline. The mean ages of the PwDs and caregivers were 82 ± 7.0 and 66 ± 12 years, respectively, and 62.8% of the PwD were female and 73.5% had access to any type of ATT. The majority had traditional equipment such as stove guards (43.3%) and social alarms (39.5%) or everyday technology, e.g. calendar support and door locks (45.3%). Multivariate regression analyses revealed that access to a social alarm was more often available for females than males, at increased age, and when the PwD lived alone, while tracking devices (14.9%) were more often accessible at lower age. Everyday technology was more often available for females, at increased age of the PwD and the caregiver, higher comorbidity, and poor IADL (instrumental activities of daily living) function. For PwDs with severe dementia, access to ATT was significantly associated with poor IADL function, having their children as the main caregiver (61.3%), and having caregivers who contributed 81–100% to their care (49.5%).

**Conclusions:**

Home-dwelling PwDs mainly had access to traditional and obligated devices, followed by everyday technology. There is unmet potential for communication, tracking, and sensing technology, especially for devices not offered by the municipalities. Gender, ages of the PwD and caregiver, cohabitation status, and physical function were the main associated factors for access to ATT.

*Trial registration*: ClinicalTrials.gov NCT04043364.

**Supplementary Information:**

The online version contains supplementary material available at 10.1186/s12911-021-01627-2.

## Background

Around 50 million people worldwide suffer from dementia, and 10 million new cases are estimated for every coming year. Most of them are women and people over 80 years of age. [1] Dementia is a chronic progressive syndrome including any brain disease causing loss of cognitive abilities such as thinking, memory, behaviour, and the ability to perform everyday activities. The signs and symptoms can be categorised as mild, moderate, and severe [1]. More severe dementia requires more comprehensive care [2], and aging populations have led to an increase in the prevalence of dementia that is challenging healthcare systems and their economy. [3, 4]

In Norway, 40% of all people receiving home care service suffer from dementia [5]. Providing care is primarily a municipal responsibility, with the aim to enable an independent and safe life for as long as possible [3]. Many family caregivers provide additional care, making them important collaborators with the home care services [6]. Because a major shortage of healthcare providers is expected, it is assumed that traditional care service delivery will not be sufficient in the future [7]. Assistive technology and telecare (ATT) may have potential for supporting home-dwelling people with dementia (PwD) and their formal and informal caregivers by improving resource utilisation and service quality [8–11].

To date, there is limited consensus on the definition and classification of ATT, and terms like ATT, welfare technology, telehealth, and telemedicine are often used synonymously [9]. In this study, ATT refers to technical devices grouped according to their purpose, while assistive technology (AT) is defined as any device or system that maintains or improves an individual’s ability to perform tasks that they would otherwise be unable to or increases the ease and safety of the tasks that are performed (e.g. memory, tracking, or communication technology) [12]. The utilisation of telecare, often defined as technology for living safely in one’s own home, differs between nations depending on their health care system structures [13, 14]. In Norway, telecare is grounded within the roots of social care and is centred on promoting safety and independence in order to enable patients to live longer in their own homes. In this context, telecare implies both personal and environmental sensors (e.g. social alarms or fall sensors) that reduce the risk at home through real-time 24-h monitoring and sensors directly addressed to the PwD (e.g. stove guards and light sensors). Real-time monitoring provides an immediate response by a base unit connected to a telephone line ('tele') monitoring service or response centre with the possibility to call for assistance ('care') when needed [14]. Telecare can be categorised in different ways because it can differ in terms of what needs to be purchased and can require different abilities from the PwDs and caregivers. One way is to make a distinction between active and passive devices. An active device (e.g. social alarm) has to be activated by the user when needed, while passive devices (e.g. fall and movement detectors) are triggered automatically [13]. Some devices have both functions, which complicates attempts at categorisation. For example, tracking technology can additionally be used as an outside used personal emergency alarm and can therefore be seen as both active and passive telecare [9]. Most passive sensor devices require installation of a pre-existing active sensor (i.e. social alarm) that is connected to a 24-h monitoring service. These sensors are therefore contemporaneously offered together with a social alarm.

In order to meet some of the key issues such as risk of falls, medication management, and cognitive impairment, national dementia strategies have been globally established to encourage the adoption and application of ATT [15]. Implementation of these strategies include assessment, recommendations, installation, monitoring, and adjustment of ATT [16]. The Norwegian government launched a national programme in 2013 for the development and implementation of ATT, with the aim to use this technology as a fully integrated part of home care services by 2020 [17]. This programme obliges the municipalities to establish systems and procedures to facilitate information, conversation, and dialogue about access and use of ATT [18].

However, policy strategies have so far not succeeded in achieving continuous and adequate access to ATT [16, 19]. Implementing ATT to support home-dwelling older people is complicated, and the adoption of especially complex ATT has been low [20, 21]. Several challenges have been be addressed. First, the diversity and variety of cognitive decline makes it challenging to ensure that the designed ATT is sufficiently flexible to serve the diversity of needs and the changing needs for the same persons because health and functional status change over time [15, 18]. Second, a significant number of caregivers and PwDs lack access to ATT due to socioeconomic status, technological literacy, and the remaining digital gap [11]. Third, the implementation is challenged by the municipality agreements with multiple involved stakeholders with constantly changing conditions and situations regarding the purchasing and servicing of ATT, as well as by a lack of national ATT standards [20, 22]. Thus, ATT not only needs to be usable and useful (fulfilling the needs of users), but also needs to be supervised by national authorities to avoid proprietary solutions and needs to be appropriately introduced by the municipalities [21].

Despite increased research in recent past years, the specific focus on ATT`s application for PwDs is limited [10], and very few devices have been brought into continued routine service [23]. Few observational studies have investigated the overall access to different ATT devices, and there is a need for knowledge about current access to different ATT devices in Norwegian home-dwelling PwDs [19]. The identification of associated factors with access to ATT might improve the implementation of ATT management in home-dwelling PwDs, and thus the main purpose for this study was to explore current access to ATT in home-dwelling PwDs in Norway. Baseline data from the ongoing LIVE@Home.Path trial were utilised to identify the different ATT devices installed in homes and the factors associated with access to ATT in general and to different ATT devices in particular.

## Method

### Study population

Between January and June 2019, the LIVE@Home.Path trial (ClinicalTrials.gov NCT04043364) screened 428 dyads of home-dwelling men and women with dementia and their informal caregivers as part of the ongoing randomised clinical trial. Participants were recruited from memory clinics at local hospitals, municipal memory teams, and through advertisements in general media such as newspapers, radio, and TV. In this stepped-wedge multicentre trial, dyads from three municipalities in Norway (Bergen, Bærum, and Kristiansand) were enrolled when the PwD met following inclusion criteria: ≥ 65 years of age, diagnosed with dementia according to a standardised protocol [24], and a Mini Mental State Examination (MMSE) [25] score of 15–26 or a Functional Assessment Scaling Tool (FAST)[26] score of 3–7. Bergen is the second-largest municipality of Norway (about 281,000 inhabitants), Bærum is the fifth largest (about 127,000 inhabitants), and Kristiansand is the sixth-largest municipality (about 92,000 inhabitants) [27].

### Design and study procedure

The 2-year LIVE trial aimed to investigate how a multicomponent intervention on Learning, Innovation, Volunteer, and Empowerment (LIVE acronym) affects resource utilisation in municipal dementia care and supports staying safer, longer, and more independently at home in a cost-effective manner. The primary outcome was resource utilisation, while secondary outcomes included the use of ATT, among others. Participants were allocated to a municipal coordinator who was responsible for contact with the participants to facilitate the implementation of the LIVE intervention [28]. All municipal coordinators went through a 2-day course on the application of selected assessment tools in addition to training on how to implement the intervention before they evaluated enrolled patients for eligibility at baseline. Data were collected at baseline, 6 months, 12 months, 18 months, and 24 months. The study protocol and more details on the study are described elsewhere [29].

To provide secure data transfer and storage on the SAFE server at the University of Bergen for research projects with sensitive data, all data were collected on tablets owned by the project group via the software SurveyJS [30]. The LIVE@Home.Path trial was selected as a pilot for the development and evaluation of this software. After approval from the principal investigator, researchers affiliated with the project were given access to the server, thus avoiding export of data and maintaining high levels of security [31].

### Data description and outcome measures

This paper explored baseline data from the LIVE@Home.Path-trial. All data on demography, medical history, and ATT were obtained through semi-quantitative questionnaires in direct conversation with the included dyads. Questions about ATT were initially directed to the PwD and then verified by their caregiver directly after.

### Primary outcomes

Two primary outcome measures were explored according to the PwD’s access to (i.e. installed) ATT at home, referring to the questions 1) “Do you have assistive technology and telecare?” and 2) “If yes, what kinds of items do you have?”. The first variable was dichotomous (yes/no), while the second variable was nominal and contained 16 different items divided into the following seven categories: passive sensor technologies (light, refrigerator, flood, fall, bed occupancy, and door sensors), obligated sensors (stove guards), active sensors (social alarms), tracking devices (with or without social alarms), everyday technology that provides support in everyday activities (timer on electronic devices, watch with memory function/voice, electronic door lock, calendar support, and electronic pill box), and video communication. Finally, the participants were given the possibility to add “other technology” if not listed. Additional file 1 provides a description of available ATT devices reported by the study population with information about devices offered by the three different municipalities, named A, B and C.

### Secondary outcomes

Continuous variables were age (PwD and caregiver), number of diagnoses, and the following five validated assessment scores: General Medical Health Rating scale (GMHR) [32], Norwegian revised MMSE-NR3 (translated from the English MMSE version)[33], FAST[26], Instrumental Activities of Daily Living (IADL) [34], and Personal Activities of Daily Living (PADL) [35] (Table 1).

**Table 1:** Validated assessment tools for use in people with dementia

Assessment tool	What is measured?	Characteristics
General medical health rating scale (GMHR)	Presence and assess severity of medical comorbidity in dementia	1–4-point scale, scored by the interviewer; 1–poor, 2-moderate, 3-good, 4–excellent health. A high score indicates high comorbidity burden
Norwegian revised mini mental state examination (MMSE-NR3)	Differentiation of severity of cognitive impairment	0–30-point scale, assessed by the interviewer and answered by the person with dementia. Categorized into four stages of severity: 0–11 = severe, 12–17 = moderate, 18–23 = mild, 24–30 = no impairment
		Six domains are covered: orientation, attention, memory, language, and visual-spatial skills. A low score indicates low cognitive function
Functional assessment staging tool (FAST)	Severity of dementia	1–7-point scale. The primary caregiver stages dementia in 7 stages; 1-normal, 2-normal ageing, 3-possible dementia, 4-mild, 5-moderate, 6-and 7-severe dementia; good reliability and validity. A high score indicates a high severity of dementia
Instrumental activities of daily living (IADL)	Physical function level for instrumental activities	8–31-point scale, answered by the person with dementia. Includes eight items for proxy assessment; use of telephone, shopping, cooking, household, doing laundry, public transport, responsibility for medication, and dealing with economy. A high score indicates poor function
Personal activities of daily living (PADL)	Physical function level for personal activities	6–30-point scale. Six items rated 1–5 for proxy assessment of personal activities such as toileting, grooming, dressing, transfer and eating. A high score indicates poor functioning. A high score indicates poor function

The following categorical variables regarding the PwD were investigated: age in tertiles (66–79/79–86/86–97 yeas), sex (male/female), residency (own flat or house/residential home/other), cohabitation status (alone/with spouse or partner), hazardous situations regarding falls (yes) and fires (yes), type of dementia (Alzheimer disease/vascular dementia/Lewy–Legene dementia/frontotemporal dementia /mixed or unspecified dementia/other types), and severity of dementia index (low/medium/high). For information about telephone use, data were collected from the IADL assessment tool and categorised by four possible answers (Operates the telephone by own initiative, looks up and dials numbers/ Dials a few well-known numbers/ Answers but does not dial/ Telephone not used). Categorical variables regarding the caregivers were explored by caregiver’s age grouped in tertiles (33–58/58–73/73–92 years), kinship (sibling/child/friend/other), living with the PwD (yes), and their contribution to the care of the PwD in five categories (1–20%/21–40%/41–60%/61–80%/81–100%). Contribution to care was self-reported and depended on the total number of caregivers for the respective PwD.

### Severity of dementia index

In line with the inclusion criteria, an index score for severity of dementia was created by combining the MMSE-NR3 and FAST assessment scores. Both variables were normally distributed. First, the scale of the MMSE-NR3 was reversely decoded to match the severity scale of the FAST score. Thereafter, the median score for each assessment tool was calculated, and the two variables were summarised. PwDs with a score equal to or above the median total score were given one point for each of the two assessment tools, and zero points were given for scores below the median. Accordingly, one point was assigned for median FAST scores ≥ 4 and for median reversed MMSE-NR3 scores ≥ 9. The total score was summed up for each PwD for a maximum score of two points and categorised into three groups of dementia severity: low (0 points), medium (1 point), and high (2 points).

### Statistics

Descriptive data are reported as means (± SD) or as numbers (percentages) and are presented for the total population of PwDs and stratified by access to AT (no/yes). Differences between groups were tested with independent samples t-tests for normally distributed continuous variables, with Mann–Whitney U-tests for nonnormally distributed continuous variables, and with Chi-squared tests for categorical variables.

Unadjusted and multivariate logistic regression analyses were applied to investigate factors associated with access to ATT in general, and multivariate analyses investigated access to different groups of ATTs, including passive, active, and obligated sensor technology, tracking devices, and everyday technology. All multivariate models were adjusted for age, sex, cohabitation status,, MMSE-NR3, FAST, GMHR, IADL, and PADL.

MMSE-NR3, FAST, GMHR, IADL, and PADL scores were calculated with hot-deck substitution of missing data. We applied Akaike’s information criterion (AIC) for model selection, and P-values < 0.05 were considered statistically significant. Statistical analyses were performed with STATA version 16.1 (StataCorp. 2019, College Station, TX) and R version 4.0.2 (R Core Team, The R Foundation for Statistical Computing, Vienna, Austria, 2020).

## Results

### Baseline characteristics

**Fig. 1:**
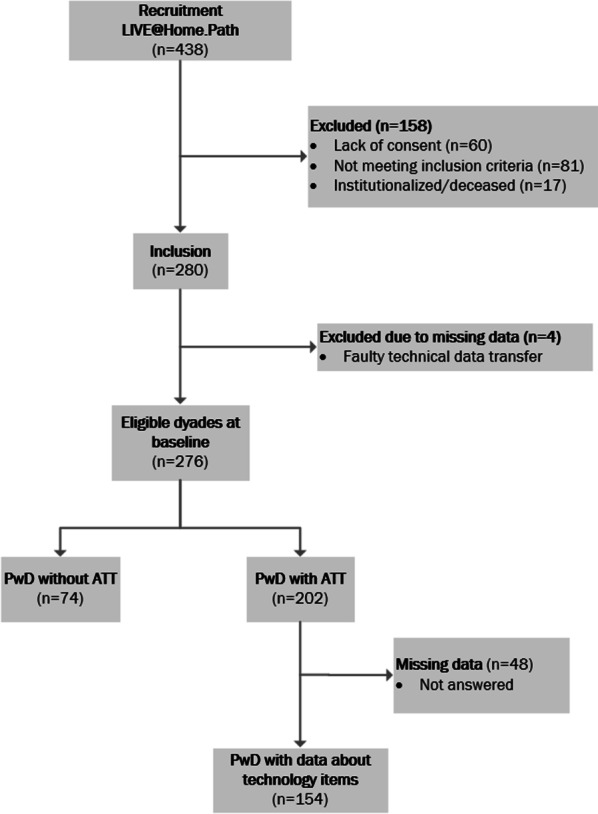
Flowchart of the study population. Illustrates included and excluded dyads of people with dementia (PwD) and caregivers, and provides an overview over who of them had access/no access to assistive technology and telecare

Figure 1 provides a flowchart of the recruited participants. During the inclusion process, 158 dyads of PwDs and caregivers were excluded due to unfulfilled inclusion criteria (n = 81), lack of consent (n = 81), or for being institutionalised or deceased (n = 17). In total 280 dyads were left to be included in the LIVE@Home.Path trial. Due to faulty technical data transfer for four PwDs, 276 dyads were left for current analyses. Among them, data from 202 PwDs with access to ATT in general and data about 154 technology items (missing data, n = 48) were explored.

Table 2 presents the baseline characteristics of the total population and differences between groups stratified by access to ATT (no/yes).

**Table 2:** Baseline characteristics of people with dementia and their caregivers for the total population and differences between groups of access to assistive technology and telecare.^a^

	Total population n = 276	Technology		*P* value^b^
		No n = 74 (26.5)	Yes n = 202 (73.5)	
Age (years)	82.1 ± 7.0	79.0 ± 6.4	83.3 ± 6.8	< .001
Age categories (years)				< .001
66–79	74.2 ± 3.6	39 (55.7)	54 (26.7)	
79–86	82.6 ± 2.0	25 (33.8)	66 (32.7)	
86–97	89.8 ± 2.3	10 (13.5)	82 (40.6)	
Sex				.001
Men	103 (37.3)	40 (54.1)	63 (31.2)	
Women	173 (62.7)	34 (45.9)	139 (68.8)	
Residency				.54
Own flat/house	264 (95.7)	72 (97.3)	192 (95.0)	
Residential home	9 (3.3)	1 (1.4)	8 (4.0)	
Other	3 (1.1)	1 (1.4)	2 (1.0)	
Cohabitation status				< .001
Alone	136 (49.3)	12 (16.2)	124 (61.4)	
Spouse/partner	135 (48.9)	60 (81.1)	75 (37.1)	
Child	5 (1.8)	2 (2.7)	3 (1.5)	
Municipality				.70
A	124 (44.9)	36 (48.6)	88 (43.6)	
B	92 (33.3)	24 (32.4)	68 (33.7)	
C	60 (21.7)	14 (18.9)	46 (22.8)	
Fall (yes)	16 (5.8)	2 (2.7)	14 (6.9)	.30
Fire (Yes)	9 (3.3)	0 (0.0)	9 (4.5)	.14
Number of diagnosis	2.57 ± 1.70	2.61 ± 1.50	2.56 ± 1.77	.39
Type of dementia				.11
Alzheimers disease	101 (36.6)	32 (43.2)	69 (34.2)	
Vascular dementia	11 (4.0)	5 (6.8)	6 (3.0)	
Lewy-Legene dementia	3 (1.1)	1 (1.4)	2 (1.0)	
Frontotemporal dementia	1 (0.4)	1 (1.4)	0 (0.0)	
Mixed/unspecified dementia	152 (55.1)	32 (43.2)	120 (59.4)	
Other types	8 (2.9)	3 (4.1)	5 (2.5)	
IADL score^c^	19.9 ± 6.1	17.6 ± 6.4	20.8 ± 5.8	< .001
Telephone use				.01
Operates by own initiative, looks up and dials numbers	78 (29.2)	30 (41.1)	48 (24.7)	
Dials a few well-known numbers	131 (49.1)	29 (39.7)	102 (52.6)	
Answers, but does not dial	42 (15.7)	9 (12.3)	33 (17.0)	
Not in use	14 (5.2)	3 (4.1)	11 (5.7)	
PADL score^d^	10.3 ± 3.3	8.6 ± 0.4	11.0 ± 0.2	< .001
GMHR score^e^	2.8 ± 0.8	2.7 ± 0.8	2.9 ± 0.8	.04
MMSE-NR3score^f^	20.7 ± 3.8	21.1 ± 3.6	20.5 ± 3.8	.21
FAST score^g^	4.2 ± 0.9	3.9 ± 0.9	4.3 ± 0.9	< .001
Severity of dementia index				.03
Low (0 poeng)	24 (8.70)	14 (18.9)	10 (5.0)	
Medium (1 poeng)	99 (36.9)	24 (32.4)	75 (37.1)	
High (2 poeng)	153 (55.4)	36 (48.7)	117 (57.9)	
Cg age (years)	66.0 ± 12.4	72.5 ± 11.2	63.6 ± 11.0	< .001
33—58	90 (53)	10 (13.5)	80 (40.8)	
58—73	90 (65)	24 (32.4)	66 (33.7)	
73—92	90 (80)	40 (54.1)	50 (25.5)	
CG sex				
Men	176 (64.7)	49 (66.2)	127 (64.1)	.77
Women	96 (35.3)	25 (33.8)	71 (35.9)	
CG kinship				< .001
Spouse	117 (43.3)	55 (74.3)	62 (30.7)	
Sibling	1 (0.4)	0 (0.0)	1 (0.5)	
Child	140 (51.6)	15 (20.3)	125 (63.3)	
Friend	2 (0.7)	1 (1.4)	1 (0.5)	
Other	11 (4.1)	3 (4.1)	8 (4.0)	
CG living with the person with dementia (yes)	125 (46.3)	58 (78.4)	67 (34.2)	< .001
CG`s contribution to care				< .001
1–20%	12 (4.5)	2 (2.7)	10 (5.1)	
21–40%	27 (9.7)	7 (9.5)	20 (10.2)	
41–60%	44 (16.4)	4 (5.4)	40 (20.4)	
61–80%	48 (17.8)	5 (6.8)	43 (21.9)	
81–100%	139 (51.7)	56 (75.7)	83 (42.3)	

^a^Continuous variables are presented as mean (± standard deviation) and categorical variables as numbers and percentages (%). Abbreviations: CG, caregiver; FAST, functional assessment scaling tool; GMHR, general medical health rating scale; IADL, instrumental activities of daily living; MMSE-NR3, Norwegian revised Mini Mental State Examination; PADL, personal activities of daily living

^b^Differences between groups of technology (yes/no) were tested with independent samples t tests for normally distributed continuous variables, Mann–Whitney U test for nonnormally distributed continuous variables and Chi squared tests for categorical variables

^c^Range 8–31, measures 8 items for proxy assessment of use of telephone, shopping, economy, public transport and household; a high score indicates poor function

^d^ range 6–30, measures 6 items 1–5 for proxy assessment of personal activities such as toileting, grooming, dressing, transfer and eating. A high score indicates poor function

^e^Range 1–4; 1– poor, 2 – moderate; 3 – good, 4 – excellent health

^f^Range 0–30, a higher score indicates more intact cognitive function

^g^Range 1–7, a high score indicates a high severity of dementia

### Total population

In total 202 PwDs (73.5%) had installed ATT at home. The mean age of PwDs was 82.1 ± 7.0 years, with a range of 66–97 years. The majority of the PwDs (62.7%) were women, and about half of them lived alone. The mean IADL and PADL score was 19.9 ± 6.1 and 10.3 ± 3.3, respectively, while the mean MMSE-NR3 and FAST score was 20.7 ± 3.8 and 4.2 ± 0.9, respectively. Most of the PwDs (55.8%) suffered from severe dementia. The minority (29.2%) were able to use the telephone properly.

The main caregivers’ mean age was 66.0 ± 12.4 years, and the majority were children (51.6%) to the PwD or were their spouse (43.3%). Almost half of the main caregivers (46.3%) lived with the PwD, and most of them (51.7%) contributed more than 80% to the PwD’s total care.

### Differences between groups

Compared to PwDs without access to ATT, those with access were older with the majority being 86–97 years old (40.6%). They were more often women (68.8%), lived alone (64%), and had a poor IADL and PADL function with mean scores of 20.8 ± 5.8 and 11.0 ± 0.2, respectively. PwDs with access to ATT were less often able to use the telephone properly, and they more often had severe dementia according to the FAST score alone with a mean score of 4.3 ± 0.9 and according to the severity of dementia index (57.9%). No differences for access to ATT between the municipalities were detected.

PwDs with access to ATT more often had younger caregivers with the majority between 33 and 58 years of age (40.8%). Their caregivers were more often their children (63.5%), less often lived together with the PwD (34.2%), and less often contributed more than 80% to their care (42.3%) compared to those without ATT.

### Technology items

Table 3 presents different devices installed among the 276 PwDs. Percentage of access is presented for the total population and among the 154 PwDs with accessible information about technology devices. When ATT was available, a mean of 2.9 ± 1.0 different items were installed.

**Table 3:** Assistive technology and telecare devices installed at home-dwelling people with dementia

Technology devices	N cases	%^a^	% ^b^
Number of different devices (mean ± SD)	2.9 ± 1.0		
Passive sensor	10	3.7	6.5
Motion	0	0	0
Refrigerator	0	0	0
Flood	1	0.4	0.6
Fall	1	0.4	0.6
Bed occupancy	3	1.1	1.9
Door	5	1.8	3.2
Obligated sensor			
Stove guard	119	43.3	77.3
Active sensor and tracking device	132	47.9	85.7
Social alarm	109	39.5	70.8
Social alarm with tracking	6	2.2	3.9
Tracking device	17	6.2	11.0
Everyday technology	125	45.3	80.9
Timer on electronic devices	25	9.1	16.2
Watch with memory function/voice	37	13.4	24.0
Electronic door lock	49	17.8	31.8
Calendar support	52	18.6	33.1
Electronic pill box	15	5.4	9.7
Door Camera	1	0.4	0.6
Video communication	0	0	0
Other technology^c^	10	3.6	6.5

^a^percentage calculated from the total population, n = 276

^b^ percentage calculated from people with dementia with information about access to technology devices, n = 154

^c^Tracking device through mobile phone, heat detector (stove), mobile application to register when the person with dementia leaves day-care center and when entering at home, night alarm when the person with dementia gets close to the entry door, and hearing aid

Among the total population, the four most-available devices were stove guards (43.3%), social alarms (39.5%), and the everyday technology devices of calendar support (18.6%) and electronic door locks (17.8%). All devices were available through the municipalities. Social alarms and stove guards are routinely offered, while everyday technology is offered upon request as part of a so-called safety package.

Among 154 PwDs with accessible information about ATT devices, the majority of PwDs had access to everyday technology (80.9%) followed by the obligated sensors (77.3%), active sensors (74.7%), tracking devices (14.9%), passive sensors (6.5%), and other technology (6.5%).Video communication aids were not reported.

### Factors associated with access to ATT in general

Regression analyses explored factors associated with access to ATT (Table 4). Several factors were associated in the unadjusted model, including age, gender, cohabitation status, FAST score, severity of dementia index, GMHR score, IADL and PADL score, and caregiver’s age, kinship, and living situation with the PwD. After multivariate adjustments, only gender, cohabitation status, and IADL score were associated with access to ATT. PwDs more often had access to ATT when they were female [odds ratio (OR) and 95% confidence interval (CI) = 2.32 (1.14–4.78), P = 0.02)], their IADL function was poor [OR (95% CI) = 1.10 (1.02–1.19), P = 0.01], and they lived alone [OR (95% CI) = 3.93 (1.33–11.6), P = 0.01].

**Table 4:** Logistic regression analysis for factors associated with access to assistive technology and telecare among 276 people with dementia^a^

	Unadjusted		Multivariate adjusted^b^	
	OR [95%CI]	*P *value	OR [95%CI]	*P *value
Age (years)				
66–79	1.00		1.00	
79–86	1.91 [1.03–3.54]	.04	1.35 [0.65–2.82]	.43
86–97	5.92 [2.73–12.85]	< .001	1.77 [0.70–4.46]	.23
Sex (female)	2.60 [1.50–4.48]	.001	2.32 [1.14–4.78]	.02
Cohabitation status				
Spouse/partner	1.00		1.00	
Alone	8.21 [4.16–16.2]	< .001	3.93 [1.33–11.6]	.01
Municipality				
A	1.00		1.00	
B	1.16 [0.63–2.12]	.63	1.40 [0.62–3.19]	.42
C	1.34 [0.66–2.74]	.42	1.02 [0.42–2.45]	.97
Fall (yes)	2.68 [0.59–12.1]	.20	1.71 [0.33–8.78]	.52
MMSE-NR3^3^ score	0.95 [0.89–1.03]	.20	0.99 [0.88–1.11]	.87
FAST^4^ score	1.80 [1.28–2.55]	.001	1.46 [0.89–2.41]	.13
Severity of dementia index				
Low	1.00		1.00	
Medium	4.38 [1.72–11.12]	.002	2.63 [0.85–8.10]	.09
High	4.55 [1.86–11.12]	.001	1.64 [0.53–5.09]	.40
GMHR^5^ score	0.68 [0.47–0.98]	.04	0.75 [0.48–1.18]	.30
PADL^6^score	0.79 [0.72–0.87]	< .001	0.90 [0.79–1.03]	.13
IADL^7^score	1.09 [1.04–1.14]	< .001	1.10 [1.02–1.19]	.01
Telephone use—operates by own initiative, looks up and dials numbers	0.44 [0.11–1.69]	0.23	0.61 [0.10–3.59]	.59
CG age (years)				
33—58	1.00		1.00	
58—73	0.34 [0.15–0.77]	.009	0.51 [0.20–1.34]	.17
73—92	0.15 [0.07–0.34]	< .001	0.34 [0.13–1.01]	.05
CG sex (men)	1.09 [0.62–1.9]	.77	1.39 [0.50–3.87]	.53
CG kinship				
Spouse	1.00		1.00	
Child	1.20 [0.19–7.41]	.84	0.44 [0.02–9.57]	.60
CG living with the person with dementia (yes)	0.14 [0.08–0.27]	< .001	1.32 [0.21–8.20]	.76
CG`s contribution to care		< .001		
1–20%	1.00		1.00	
21–40%	0.54 [0.09–3.12]	.49	1.23 [0.13–11.46]	.87
41–60%	2.00 [0.32–12.51]	.46	5.04 [0.57–50.75]	.14
61–80%	1.72 [0.29–10.18]	.55	4.26 [0.47–38.50]	.20
81–100%	0.30 [0.06–1.40]	.13	1.81 [0.23–14.11]	.56

^a^Abbreviations: CG, caregiver; CI, confidence interval; FAST, functional assessment scaling tool; GMHR, general medical health rating scale; IADL, instrumental activities of daily living; MMSE-MR3, Norwegian revised Mini Mental State Examination; OR, odds ratio; PADL, personal activities of daily living

^b^Logistic regression analyses adjusted for age (PwD and cg), sex, cohabitationliving status, MMSE-NR3, FAST, GMHR, IADL, and PADL.GMHR, IADL, PADL, MMSE-NR3 and FAST

In subgroup analysis by the severity of dementia index (Additional file 2), access to ATT was associated with worse IADL function (P < 0.001) and better PADL function (P < 0.001), the caregiver’s kinship to the PwD (child (61.3%), P = 0.006), and a higher contribution to care by the caregivers (81–100% contribution (49.5%), P = 0.02) compared to those without access to ATT. Additional file 3 presents a regression plot for subgroup analysis by MMSE-NR3 score stratified by teriles of age. Access to ATT decreased with higher MMSE score (i.e. less severe dementia) between PwDs in the age groups of 66–77 and 89–97 years.

### Factors associated with access to different kinds of ATT

Multivariate regression analyses (Table 5) revealed that the gender and age of the PwD were associated with access to a social alarm [gender: OR (95% CI) = 2.48 (1.21–5.10), P = 0.01; age: OR (95% CI) = 1.13 (1.07–1.19), P < 0.001], stove guard [gender: OR (95% CI) = 0.45 (0.24–0.84), P = 0.01; age: OR (95% CI) = 1.07 (1.02–1.12), P = 0.007], and everyday technology [gender: OR (95% CI) = 1.96 (1.04–3.70), P = 0.04; age: OR (95% CI) = 1.07 (1.02–1.12), P = 0.01)]. Tracking devices were only associated with increased age of the PwD [OR (95% CI) = 0.88 (0.82–0.96), P = 0.004]. Cohabitation status was associated with access to a social alarm [OR (95% CI) = 3.96 (1.52–10.3), P = 0.0005]. Age of the caregiver was associated with access to a stove guard [OR (95% CI) = 0.96 (0.93–0.99), P = 0.002] and everyday technology [OR (95% CI) = 0.95 (0.92–0.98), P = 0.002]. Comorbidity (GMHR) [OR (95% CI) = 1.54 (1.05–2.26), P = 0.03] and I-ADL [OR (95% CI) = 1.10 (1.02–1.18, P = 0.008] were associated with everyday technology. Access to passive sensor technology was not associated with any investigated factors, and severity of dementia alone was not associated with access to any of the ATT items.

**Table 5:** Factors associated with access to different assistive technology and telecare items in home-dwelling persons with dementia.^a^

	Sensor devices						Tracking device^c^ (n = 23)		Everyday devices^d^ (n = 124)	
	Passive^b^ (n = 11)		Active (social alarm) (n = 109)		Obligated (stove guard) (n = 119)					
	OR [95%CI]	*P *	OR [95%CI]	*P *	OR [95%CI]	*P *	OR [95%CI]	*P *	OR [95%CI]	*P *
Sex (female)	4.16 [0.41–42.6]	.23	2.48 [1.21–5.10]	.01	2.21 [1.18–4.11]	.01	1.52 [0.54–4.30]	.43	1.96 [1.04–3.70]	.04
Age	0.98 [0.85–1.13]	.76	1.13 [1.07–1.19]	< .001	1.07 [1.02–1.12]	.007	0.88 [0.82–0.96]	.004	1.07 [1.02–1.12]	.007
Cohabitation status										
Spouse/partner	1.00		1.00		1.00		1.00		1.00	
Alone	3.05 [0.14–65.2]	.48	3.96 [1.52–10.3]	.005	1.49 [0.64–3.48]	.36	1.31 [0.28–6.19]	.73	1.44 [0.59–3.48]	.42
Fall (yes)	NA		1.53 [0.46–5.12]	.49	NA		NA		0.92 [0.30–2.82]	.88
Severity of dementia index										
Low	1.00		1.00		1.00		1.00		1.00	
Medium	1.46 [0.35–6.16]	.60	1.42 [0.35–5.80]	.63	3.49 [1.00–12.2]	.05	1.52 [0.36–6.51]	.57	2.51 [0.71–8.89]	.15
High	2.57 [0.61–10.9]	.20	2.63 [0.64–10.7]	.18	2.80 [0.79–9.90]	.11	2.01 [0.42–9.58]	.38	2.94 [0.82–10.5]	.10
GMHR	0.89 [0.57–1.37]	.58	0.90 [0.60–1.35]	.60	1.11 [0.76–1.60]	.58	1.53 [0.80–2.94]	.20	1.54 [1.05–2.26]	.03
PADL	0.95 [0.74–1.21]	.66	1.03 [0.91–1.15]	.67	0.98 [0.89–1.09]	.78	0.90 [0.76–1.05]	.19	1.00 [0.90–1.11]	.98
IADL	1.03 [0.87–1.20]	.76	1.06 [0.98–1.14]	.13	1.02 [0.96–1.09]	.53	1.01 [0.92–1.13]	.78	1.10 [1.02–1.18]	.008
Telephone use—operates by own initiative, looks up and dials numbers	0.26 [0.05–1.38]	.11	0.27 [0.06–1.32]	.11	5.41 [0.55–53.4]	.15	0.15 [0.01–4.04]	.26	0.29 [0.07–1.28]	.10
CG age	1.00 [0.96–1.03]	.83	1.00 [0.96–1.03]	.78	0.96 [0.93–0.99]	.02	0.99 [0.94–1.05]	.79	0.95 [0.92–0.98]	.002
CG sex (men)	1.01 [0.18–5.78]	.99	1.11 [0.56–2.22]	.76	1.05 [0.55–1.98]	.87	0.46 [0.12–1.74]	.25	0.85 [0.44–1.64]	.63
CG living with the PwD (yes)	0.28 [0.06–1.42]	.13	0.36 [0.08–1.69]	.20	0.49 [0.12–2.02]	.32	0.94 [0.10–8.71]	.96	0.15 [0.01–1.73]	.13
CG`s contribution to care										
1–20%	1.00		1.00		1.00		1.00		1.00	
21–40%	1.33 [0.23–7.79]	.76	0.65 [0.10–4.15]	.65	0.34 [0.07–1.64]	.18	0.41 [0.02–8.44]	.65	0.91 [0.18–4.64]	.91
41–60%	0.67 [0.13–3.47]	.64	0.37 [0.07–2.10]	.26	0.94 [0.22–4.05]	.93	1.28 [0.11–14.7]	.84	0.94 [0.21–4.20]	.94
61–80%	0.46 [0.09–2.32]	.35	0.26 [0.05–1.43]	.12	0.59 [0.14–2.48]	.47	1.09 [0.10–12.5]	.94	0.69 [0.16–3.02]	.62
81–100%	0.67 [0.13–3.31]	.62	0.41 [0.07–2.20]	.30	0.54 [0.13–2.21]	.39	0.83 [0.07–10.1]	.88	0.58 [0.13–2.53]	.47

^a^n = 276. Access to one of the devices or one ATT group did not mutually exclusive the access to others. P-value calculated by logistic regression, adjusted for age (PwD + cg), sex (PwD), living statuscohabitation status, MMSE-NR3, FAST, GMHR, IADL, and PADL.GMHR, IADL, PADL, MMSE-NR3, and FAST. Abbreviations: CI, confidence interval; CG, caregiver; FAST, functional assessment staging tool; GMHR, general medical health rating; IADL, instrumental activities of daily living; MMSE-NR3, Norwegian revised Mini Mental State Examination; OR, odds ratio; P, P-value; PADL, personal activities of daily living; PwD, pereson with dementia

^b^6 items; light sensor, refrigerator sensor,, flood-, fall-, bed occupancy- and door sensor

^c^Tracking device and social alarm with tracking device

^d^Calendar support, door camera, electronic door lock, electronic pill box, timer on electronic device, and watch with memory function/voice

## Discussion

### Principal results

Our primary aim was to explore current access to ATT in home-dwelling PwDs in Norway. We found that 74% of PwDs had access to ATT, but these were mostly traditional devices such as stove guards and social alarms, while passive sensors and tracking technology were limited and communication devices were not used at home. The gender and age of the PwD were the main associated factors with access to ATT overall, while access to everyday technology was additionally associated with comorbidity, physical function, and age of the caregiver. In different stages of dementia, physical function and the caregiver’s contribution to care were associated with the general access to ATT. These findings are of key importance for stakeholders, politicians, and homecare services because municipalities are in need of clear research-based recommendations from the government regarding the selection and implementation of valid and effective technologies. Further, there is a need for individual education programmess on ATT for PwDs, their caregivers, and the home-care staff who support these families.

### Access to ATT devices

Policy strategies have so far not succeeded in achieving continuous and adequate access to ATT both because there is a lack of national standards and because of the presence of multiple proprietary solutions and rapid technological developments with the steady introduction of new devices and solutions [16, 19].

Our findings reflect the previously described challenge of providing complex and novel ATT (i.e. passive sensor items, tracking devices, and communication aids) to home-dwelling PwDs, [13, 18, 20] whereas traditional devices such as stove guards and social alarms are more often available and are more often an integrated part of living environments for elderly people.[3, 36, 37] The municipalities included in this study are supposed to offer a social alarm and stove guard to all people > 75 years (municipality C), > 85 years (municipality A), or upon request (municipality B). Additionally, as part of their consultation visits they offer a so-called “safety package”, which depending on the municipality includes certain passive sensor devices, tracking devices, and everyday technology. These devices are either free or can be purchased for little cost [38]. Because these devices, in prticluar social alarm and stove guard were available in our population, this emphasises the influence of the municipalities regarding their consultation and purchasing of ATT.

The installation of passive sensor devices requires a preinstalled social alarm. Despite the high occurrence of social alarms, it can be questioned why only 3.7% of our participants reported having passive sensor devices installed. The purchase of sensor devices requires a mapping visit made by the healthcare workers, in contrast to the social alarm. It can therefore be suggested that sensor devices are less easily provided because the purchase involves more time from the formal caregivers. Moreover, sevreal sensor devices require the additional involvement of an informal caregiver in order to receive the incoming alarm sent by the support centre. Among PwDs with available technology, 34.2% lived together with their main caregivers. A qualitative study from the UK revealed that family caregivers play a crucial role in supporting older people's decisions to adopt and engage with ATT devices. [39] Even though we did not have data on use of ATT, it can be speculated as to whether the caregivers have an influencing role in the purchasing and adaption of ATT.

To evaluate a crucial measure to improve ATT for home dwelling persons with complex condition including dementia is a validated standard to access the association between the disease, age, gender and implemented ATT. An important publication by Asghar et al. (2019) demonstrated that it is possible to explore different needs and supporting technology with positive effects by the use of a quantitative questionnaire [40]. This tool measures area of need such as operational-, physical- and social support. In the past few years, simple communication aids have been developed especially directed to elderly people to prevent loneliness [41]. However, no communication aids were installed among our PwDs. Possible reasons may be that these devices are not provided by the municipalities and have to be purchased privately. Moreover, there are high starting costs, and not every PwD or their caregiver might have the resources to afford these devices. A recent randomised clinical trial from the UK explored routines of ATT practice and the systems in place to deliver ATT to 495 home-dwelling PwDs. The study revealed that 53% of the recommended technology was not installed, and assessment recommendations were routinely disregarded at the point of installation [16]. Moreover, successful implementation of telecare depends on how each user accepts and utilises the technology. When people feel that ATT does not improve their situation, they will stop using it.[10, 11]. Additionally, new technology requires sufficient Internet connection and increasingly involves digital components, such as internet-based remote monitoring [42], mobile technology, and smartphones [43]. It has been reported that increasing numbers of low-cost novel devices (e.g. sensor and tracking technology) and free mobile applications can be useful for both caregivers and PwDs. Notably, most of these require a smartphone, leaving out those with low technological literacy [11]. The majority of our PwDs were no longer familiar with use of a telephone and thus probably not with a smartphone either. PwDs with caregivers of lower age more often had access to ATT, whereas PwDs who lived with their spouse less often had ATT. We suspect that low access to novel ATT might be caused by the lack of technological literacy and therefore a low interest for ATT by the PwDs and their caregivers.

### Factors associated with access to ATT

Our results underline the importance of distinguishing between different ATT devices when factors for access to ATT are investigated. For instance, increased age of the PwD was associated with access to ATT independent of the type of technology (except for social alarm), while only everyday technology was associated with comorbidity and physical function.

The increased access to ATT when PwDs lived alone may be explained by the fact that PwDs with access to ATT more often had younger caregivers with the majority 33–58 years of age (40.8%) and who contributed less often more than 80% to their care (42.3%), compared to those who did not had access to ATT. Thus, younger caregivers might have a higher interest in ATT than the older generation of spouses to the PwDs. Moreover, they may have used ATT as a care substitute to help ease some of their caregiving tasks, as described in a recent study [44]. This further may explain why women more often had access to ATT because they mostly lived alone (63%) and their caregivers more often were their children (66.7%) (data not shown). Additionally, women normally live longer than men [45], and there is a higher proportion of females suffering from dementia.

In a previous study, caregivers experienced calendar reminders as a practical aid in order to support cognitive and emotional efforts, but little awareness among PwDs of electronic AT was reported [46]. Thus, it can be suggested that the increased access to everyday technology is not a result due to the PwD’s age or poor physical condition, but is influenced by the caregiver, especially when they are younger.

Lower age of the PwD increased the possibility for having access to tracking devices. This is in accordance with a study from the Netherlands exploring the use of tracking devices among people with early stages of dementia and their informal caregivers. Compared to our study, their population of PwDs was younger (mean age 73 years), more often male (83%), had less severe dementia, and were apparently able to use a mobile phone [47]. Because we did not find any association with gender or severity of dementia, our results may be comparable.

Sensor technology has been reported to make life easier and safer for PwDs and their families [48]. However, active sensor technology such as social alarms has been reported to be challenging for people with cognitive decline, and passive sensor technology may therefore be a solution [10]. In this study, access to technology increased for PwDs over 86 years of age and with more severe dementia (57.9%), and it can therefore be questioned why only 6.9% of them had access to passive sensor technology. Our results underline the importance of identifying the best ATT at the right time so as to be able to take full advantage of the potential of each technology.

### Strengths and limitations

Strengths of this study are the multi-centre cohort with data from over 540 home-dwelling male and female PwDs and their caregivers with information regarding access to a variety of ATT devices. The study cohort reflects the ratio of gender and age of PwDs in the general population, and thus the results might be generalisable. However, some limitations must be addressed. First, in Norway a stove guard is obligatory in all houses built after 2010 [49]. Thus it could be claimed that these device in particular are not self-chosen by the PwD or the caregiver, but are imposed by the municipalities. The included PwDs had a mean age of 82 years, with the assumption that the majority lived in residences built before 2010. Thus, the stove guard should have been offered to most of them and a higher percentage than 43.3% would have been expected. This might indicate that the access to stove guards may not be enforced after all. Second, data on other technology devices than social alarms and stove guards were limited, and thus we could not undertake a full analysis of all technology items individually. Therefore, technology groups were mainly clustered by practical considerations, and other possible group formations might have led to different results. The low occurrence of sensor technology (6.9%) and the low statistical power may have caused the lack of associations between different factors and these devices. Third, even though caregivers verified the PwDs` information about accessible technology, novel and less common devices may have been underreported due to lack of knowledge and less awareness of their existence. Further, we have included nine variables for adjustment in the analyses even though the number of people with dementia without ATT only was 74. We are aware about that this might have affect the statistical robustness of the modell. We also made the decision to use the AIC for model selection. Another model might have given us another satisfying result, and we are aware of that the second-order corrected AIC (AICC) may have given us a deeper focus. Finally, we did not have data on the actual request for and the use of ATT. Thus, the high access to some devices, such as social alarms, may not reflect the actual use, and the associated factors might be a result of overreported access to certain ATT. Therefore, our findings must be interpreted with caution.

## Conclusions

The findings illustrate the continuing challenge regarding access to complex and novel ATT in home-dwelling PwDs as a possible solution for staying safely and independently in their own homes for as long as possible. Being female, of increasing age, living alone, and with comorbidity and low physical function were key factors for access to ATT. Notably, these depended on the type of technology. Because access to ATT clearly requires different abilities by the PwD and caregiver, these results may help health-care workers in future mapping visits to make appropriate choices regarding ATT for home-dwelling PwDs. Moreover, access to ATT seems to be influenced by the choice of supply in the municipalities and by a lack of national standardisation. There is potential for sensor technology, appropriate communication devices, and tracking devices in addition to other novel ATT beyond current agreements between stakeholders and the municipalities.

Further studies might explore a) individually to whom, when, and what ATT devices should be provided during the course of dementia, b) to what extent the reason for low access to novel devices is caused by the limited offerings through the municipalities or by low interest for ATT among the PwDs and caregivers, and they might c) investigate mapping policies among the municipalities and whether programmes offering implementation of more novel devices such as passive sensor devices are more successful.

## Supplementary Information

**Additional file 1.** Description on available assistive technology and telecare devices among 154 people with dementia with accessible data, and information over devices offered by the three municipalities A-C.

**Additional file 2.** Baseline characteristics of 202 people with dementia with access to assistive technology and telecare, stratified by severity of dementia.

**Additional file 3.** Regression plot. Illustrates the association between assistive technology and telecare with dementia severity in home-dwelling people with dementia. The y-axeis demonstrates the probability (OR) and 95% confidence interval of having installed assistive technology and telecare , while the x-axis presents the severity of dementia (referring to the MMSE total score). The model is stratified by tertiles of age groups and adjusted for sex. The p-value is calculated by comparing agegroups 79-86 yeras and 86-97 years with the agegroup 66-79 years.

## Data Availability

The datasets used and/or analysed during the current study are available from the corresponding author on reasonable request.

## Abbreviations

AT: Assistive technology
ATT: Assistive technology and telecare
CG: Caregiver
CI: Confidence interval
FAST: Functional assessment staging tool
GHMR: General Medical Health Rating
IADL: Instrumental activities of daily living
MMSE-NR3: Norwegian revised mini mental state examination
OR: Odds ratio
PADL: Personal activities of daily living
PwD: People with dementia
