# Supplementary material for: Factors associated with access to assistive technology and telecare in home-dwelling people with dementia: baseline data from the LIVE@Home.Path trial
Source: BMC Med Inform Decis Mak. 2021 Sep 15;21:264. doi: 10.1186/s12911-021-01627-2 (PMC8442311; doi:10.1186/s12911-021-01627-2)
Supplement: Supplementary file 1 — Additional file 1. Description on available assistive technology and telecare devices among 154 people with dementia with accessible data, and information over devices offered by the three municipalities A-C. [file 12911_2021_1627_MOESM1_ESM.docx]

# Additional Files

**Additional file 1** - Description on available assistive technology and telecare devices among 154 people with dementia with accessible data, and information about devices offered by the municipalities (A-C).

| **Sensor technology** | **Technology device** | **Description of assistive technology and telecare devices** | **Municipalities^a^** | | |
| --- | --- | --- | --- | --- | --- |
|  |  |  | **A** | **B** | **C** |
|  | Fall sensor^e^ | A passive sensor that is triggered at heavy fall by accelerometer. The device is part of an integrated telecare system and is connected to a monitoring center. | X^d,f^ | X^d,f^ | X^d,f^ |
|  | Flood sensor | A passive sensor that is designed to detect unwanted water on the floor. Provides an alert in time to allow the prevention of water leakage. |  |  |  |
|  | Bed occupancy sensor^e^ | A passive sensor placed under the bed sheets sending an alert immediately when the patient exists the bed. Alternatively, the alarm can be set to send an alert only when a person does not return to bed within a reasonable time. The sensor is part of an integrated telecare system. | X^d,f^ | X^d,f^ | X^d,f^ |
|  | Door sensor^e^ | A passive sensor that detects when someone leaves through the fromt door while an alert is sent to a connected monitoring service. | X^d,f^ | X^d,f^ | X^d,f^ |
|  | Stove guard | A passive sensor that automatically turns off the stove if a situation becomes dangerous. The device is a legal requirement in all Norwegian homes built after 2010. | x^c^ | x^f^ | x^b^ |
|  | Social alarm | Active sensor device the person can carry with them as wristwatch or necklace. Requires connection to a monitoring service offered by the municipality. Triggers an alarm by active press on an integrated alarm button. Functions only nearby the monitoring router installed at home, and do not work when the person with the sensor is outside the home. | x^c^ | x^d^ | x^b^ |
| **Tracking devices** | Tracking device | Passive electronic security device which monitors the location of a person device, connected to a unit e.g., GPS (Global Positioning System) to allocate a person wearing this device. |  | x^f^ |  |
|  | Tracking device with social alarm | A combination of active/passive device connected to a GPS unit together with an installed social alarm in connection to either a monitoring service or the caregiver’s mobile phone. |  |  |  |
| **Everyday technology** | Door Camera | A camera installed outside the front door to show who is ringing the doorbell. |  |  |  |
|  | Timer on electronic devices | A control device that after a predetermined time interval, automatically starts or stops an electronic device. |  |  |  |
|  | Watch with memory function | A watch that helps to remember preinstalled appointments through an audible voice alarm. |  |  |  |
|  | Electronic door lock | Physical and electronic locking control that enables the patient or healthcare worker to enter the home when needed, without the need of a traditional key. | x | x^f^ | x |
|  | Calendar support | Display digital the time and date of the current day. | x | x | x |
|  | Electronic pill box dispenser | Electronic pill box that triggers an alarm and releases medication at a preinstalled time. | x | x^f^ |  |

^a^Muncipality (%); A: 35.2%; B:36.5%; C:38.0%.

^b^Offered to all people with dementia >75 years who claim for this device, without a required mapping visit.

^c^Offered to all people with dementia >85 years who claim for this device, without a required mapping visit.

^d^Offered to all people with dementia without a required mapping visit.

^d^Requires an installed social alarm and is connected to a 24-hours monitoring service driven by the municipalities.

^f^Part of a safety package offered by the municipalities.
