# Supplementary material for: Factors associated with access to assistive technology and telecare in home-dwelling people with dementia: baseline data from the LIVE@Home.Path trial
Source: BMC Med Inform Decis Mak. 2021 Sep 15;21:264. doi: 10.1186/s12911-021-01627-2 (PMC8442311; doi:10.1186/s12911-021-01627-2)
Supplement: Supplementary file 2 — Additional file 2. Baseline characteristics of 202 people with dementia with access to assistive technology and telecare, stratified by severity of dementia. [file 12911_2021_1627_MOESM2_ESM.docx]

**Additional file 2** Baseline characteristics of 202 people with dementia with access to assistive technology and telecare, stratified by the severity of dementia index.^a^

|  | **Severity of dementia Index** | | | **P-value^b^** |
| --- | --- | --- | --- | --- |
|  | **Low**  n= 10 (5.0) | **Middle**  n= 75 (37.1) | **Severe**  n= 117 (57.9) |  |
|  |  |  |  |  |
| **Age (years)** | 81.9 (8.20) | 84.2 (6.52) | 82.8 (6.88) | 0.35 |
| **Age categories (years)** |  |  |  | 0.88 |
| 66-79 | 3 (30.0) | 18 (24.0) | 33 (28.2) |  |
| 79-86 | 3 (30.0) | 23 (30.7) | 40 (34.2) |  |
| 86-97 | 4 (40.0) | 34 (45.3) | 44 (37.6) |  |
| **Sex (men)** | 3 (30.0) | 25 (33.3) | 35 (29.9) | 0.88 |
| **Residency** |  |  |  | 0.96 |
| Own flat/house | 10 (100.0) | 71 (94.7) | 111 (94.9) |  |
| Residential home | 0 (0.0) | 3 (4.0) | 5 (4.3) |  |
| Other | 0 (0.0) | 1 (1.3) | 1 (0.9) |  |
| **Cohabitation status** |  |  |  | 0.45 |
| Alone | 7 (70.0) | 50 (66.7) | 67 (57.3) |  |
| Spouse/partner | 3 (30.0) | 25 (33.3) | 47 (40.2) |  |
| Child | 0 (0.0) | 0 (0.0) | 3 (2.6) |  |
| **Fall (yes)** | 1 (10.0) | 3 (4.0) | 10 (8.5) | 0.45 |
| **Fire (Yes)** | 1 (10.0) | 3 (4.0) | 5 (4.3) | 0.68 |
| **Number of diagnosis** | 2.0 (1.5) | 2.6 (2.0) | 2.6 (1.7) | 0.58 |
| **Type of dementia** |  |  |  | 0.11 |
| Alzheimers disease | 3 (30.0) | 22 (29.3) | 44 (37.6) |  |
| Vascular dementia | 1 (10.0) | 2 (2.7) | 3 (2.6) |  |
| Lewy-Legene dementia | 1 (10.0) | 1 (1.3) | 0 (0.0) |  |
| Frontotemporal dementia | 5 (50.0) | 48 (64.0) | 67 (57.3) |  |
| Mixed/unspecified dementia | 0 (0.0) | 2 (2.7) | 3 (2.6) |  |
| **MMSE-NR3^c^ score** | 24.0 (1.7) | 23.5 (3.0) | 18.1 (2.5) | <0.001 |
| **FAST^d^ score** | 2.80 (0.4) | 4.1 (0.7) | 4.6 (0.8) | <0.001 |
| **GMHR^e^ score** | 0.80 (0.4) | 0.6 (0.5) | 0.6 (0.5) | 0.47 |
| **IADL^f^score** | 14.7 (5.7) | 19.6 (5.4) | 22.0 (5.2) | <0.001 |
| **PADL^g^ score** | 21.6 (2.4) | 19.3 (2.8) | 17.8 (4.1) | <0.001 |
| **Cg age (years)** | 62.58 (12.9) | 64.0 (12.0) | 63.4 (12.0) | 0.92 |
| **Cg sex (men)** | 3 (30.0) | 30 (40.0) | 38 (33.6) | 0.62 |
| **Kinship** |  |  |  | 0.006 |
| Spouse | 3 (30.0) | 21 (28.0) | 38 (34.2) |  |
| Sibling | 1 (10.0) | 0 (0.0) | 0 (0.0) |  |
| Child | 6 (60.0) | 50 (66.7) | 68 (61.3) |  |
| Friend | 0 (0.0) | 1 (1.3) | 0 (0.0) |  |
| Other | 0 (0.0) | 3 (4.0) | 5 (4.5) |  |
| **Cg living with the PwD (yes)** | 7 (70.0) | 51 (68.0) | 71 (64.0) | 0.81 |
| **Cg`s contribution to care** |  |  |  | 0.02 |
| 1-20% | 0 (0.0) | 9 (12.2) | 1 (0.9) |  |
| 21-40% | 0 (0.0) | 7 (9.5) | 12 (10.8) |  |
| 41-60% | 3 (30.0) | 15 (20.3) | 22 (19.8) |  |
| 61-80% | 4 (40.0) | 18 (24.3) | 21 (18.9) |  |
| 81-100% | 3 (30.0) | 25 (33.8) | 55 (49.5) |  |

^a^The index was defined by merging the total scores of MMSE and FAST (low =0 poeng; middle=1 poeng; severe=2 poeng). Abbreviations: Cg, caregiver; FAST, functional assessment scaling tool; GMHR, general medical health rating scale; IADL, instrumental activities of daily living; MMSE-NR3, norwegian revised mini-mental state examination; PADL, personal activities of daily living; and PwD, people with dementia.

^b^ Unadjusted logistic regression analyses

^c^range 0-30, a higher score indicates more intact cognitive function.

^d^range 1-7, a high score indicates high functional impairment.

^e^range 1-4; 1– poor, 2 – moderate; 3 – good, 4 – excellent health

^f^ range 6-30, a high score indicates poor function.

^g^range 8-31, a high score indicates poor function.
