# Supplementary material for: Factors associated with access to assistive technology and telecare in home-dwelling people with dementia: baseline data from the LIVE@Home.Path trial
Source: BMC Med Inform Decis Mak. 2021 Sep 15;21:264. doi: 10.1186/s12911-021-01627-2 (PMC8442311; doi:10.1186/s12911-021-01627-2)
Supplement: Supplementary file 3 — Additional file 3. Regression plot. Illustrates the association between assistive technology and telecare with dementia severity in home-dwelling people with dementia. The y-axeis demonstrates the probability (OR) and 95% confidence interval of having installed assistive technology and telecare , while the x-axis presents the severity of dementia (referring to the MMSE total score). The model is stratified by tertiles of age groups and adjusted for sex. The p-value is calculated by comparing agegroups 79-86 yeras and 86-97 years with the agegroup 66-79 years. [file 12911_2021_1627_MOESM3_ESM.docx]

**Additional file 3** – Regression plot


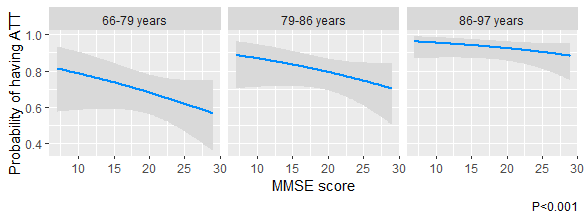
Illustrates the association between assistive technology and telecare with dementia severity in home-dwelling people with dementia. The y-axis demonstrates the probability (OR) and 95% confidence interval of having installed assistive technology and telecare, while the x-axis presents the severity of dementia (referring to the MMSE total score). The model is stratified by tertiles of age groups and adjusted for sex. The p-value is calculated by comparing age groups 79-86 years and 86-97 years with the age group 66-79 years.
